# Nursing management decisions in fever: a mixed-methods approach to understanding

**DOI:** 10.1016/j.ijnsa.2026.100486

**Published:** 2026-01-07

**Authors:** Lu-Yen A Chen, Tonks N Fawcett, Colin Chandler, Tzu-Wen Weng

**Affiliations:** a Institute of Clinical Nursing, College of Nursing, National Yang Ming Chiao Tung University, Taipei, Taiwan; b Nursing Studies, The School of Health in Social Science, The University of Edinburgh, Edinburgh, UK

**Keywords:** Fever management, Decision making, Mixed-methods, Fever phobia, Body temperature changes, Body temperature regulation

## Abstract

**Background:**

Fever is a common symptom among hospitalized patients and often triggers nursing interventions. Although clinical guidelines recommend the use of antipyretics primarily to alleviate patient discomfort rather than to normalize temperature, both pharmacological and non-pharmacological treatments remain routinely employed. However, the rationale behind these interventions in adult care settings remains poorly understood.

**Aim:**

This study aims to explore how nurses manage fever in adult patients and examine the rationale underpinning their clinical decisions in fever management.

**Methods:**

A sequential explanatory mixed-methods design was employed. Quantitative data on nurses’ fever management and decision-making for patients with fever were collected via an online survey of 177 registered nurses in Scotland. Qualitative data on nurses’ rationale and experience in managing fever were obtained through open-text responses in the online survey and five follow-up interviews. Thematic analysis and descriptive statistics were integrated to interpret findings.

**Results:**

Independent nursing judgement was the most frequently cited rationale for fever intervention (49.2 %). However, according to the qualitative finding, clinical decisions were predominantly influenced by habitual routines, institutional norms, and risk-averse thinking. Participants frequently initiated interventions as part of the Sepsis Six protocol, even in the absence of confirmed infection. Fever phobia, defined as exaggerated perceptions of fever-related harm, was evident in both interview and questionnaire data. Comfort was commonly cited as a justification for treatment, yet few participants reassessed patient comfort after administering interventions.

**Conclusion:**

Nurses’ fever management is influenced more by embedded routines and clinical culture than by formal knowledge or guideline adherence. Fever phobia and Sepsis Six pressures can contribute to unnecessary intervention, highlighting the need for reflective education and systems-level support for evidence-based practice.

The result of this study illustrated the potential for overtreatment in fever care and provides actionable insights to support evidence-informed decision-making in nursing practice. Future initiatives should focus on challenging fever phobia, supporting critical reflection, and aligning practice with current evidence through targeted educational strategies.

**What is already known about the topic:**

●Evidence-based guidelines recommend treating fever only when it causes discomfort or reaches hyperpyrexia levels, yet routine antipyretic use remains common in clinical practice.

●“Fever phobia” is known to influence clinical behaviour potentially contributed to overtreatment. The Sepsis Six protocol in known to positively influence clinical behaviour, in relation to fever, but potentially make contribute to overtreatment.

**What this paper adds:**

●Fever management by nurses is shaped primarily by clinical habit, risk aversion, and institutional framing rather than individual knowledge or patient-reported discomfort.

●The decision to intervene is often driven by defensive practices and the perceived need to act, rather than critical appraisal or guideline adherence.

●Education and practice should focus on reflective decision-making and reducing overtreatment.

Alt-text: Unlabelled box

## 1 Introduction

Fever is a common and clinically significant manifestation encountered in hospitalised patients, often signalling underlying infection, inflammation, or other systemic stressors (Barichello et al., 2022, Haidar and Singh, 2022). Defined as a core body temperature exceeding the normal physiological range of 36°C to 37.5°C, fever serves as a diagnostic hallmark and a physiological defence mechanism (Balli et al., 2023, Barichello et al., 2022, Fukuda and Nishida, 2020, Mackowiak et al., 2021). Despite its protective role in enhancing immune responses, the management of fever remains inconsistent in clinical practice and is frequently influenced by misconceptions (Fukuda and Nishida, 2020, Kasbekar et al., 2021, Mackowiak et al., 2021, Sund Levander and Tingström, 2018).

The rationale for fever management is widely debated. On the one hand, fever may exacerbate the condition of vulnerable patients, particularly those with sepsis, neurological injury, or cardiovascular compromise, by increasing metabolic demands, heart rate, and oxygen consumption (Kasbekar et al., 2021, Kimura et al., 2023, Mackowiak et al., 2021, Peters et al., 2019, Sund Levander and Grodzinsky, 2024). On the other hand, fever has been shown to support host defence mechanisms by inhibiting microbial replication and activating immune pathways (Evans et al., 2015, Haidar and Singh, 2022, Wang et al., 2020). Emerging evidence suggests that febrile patients with infections may have better outcomes than their febrile counterparts (Nicolas et al., 2023). Consequently, indiscriminate antipyretic use, particularly when not aimed at relieving discomfort, can obscure diagnostic clues, delay treatment, and potentially prolong recovery (Peters et al., 2019).

Clinical guidelines, including those from the National Institute for Health and Care Excellence, recommend the use of antipyretics primarily to manage discomfort rather than to routinely reduce temperature (Clark et al., 2024, Kelly et al., 2016, NICE, 2019, Smith et al., 2022, van de Maat et al., 2020). Nonetheless, both pharmacological and non-pharmacological treatments are frequently applied in practice (Clark et al., 2024, Kelly et al., 2016, van de Maat et al., 2020). Nurses, as frontline health caregivers, are often responsible for initiating these interventions based on their own judgement or institutional norms. While this autonomy supports responsive care, previous research has highlighted inconsistencies in nurses’ knowledge of fever physiology and management (Kelly et al., 2016, Nicolas et al., 2023, van de Maat et al., 2020). Factors influencing these decisions may include patient distress, fear of febrile seizures, misconceptions about fever-related brain damage, and habitual practices observed during clinical training (Kelly et al., 2016, Nicolas et al., 2023, van de Maat et al., 2020).

Management strategies typically involve either pharmacological or non-pharmacological interventions or a combination of both (Barathan, 2024, Hammond and Boyle, 2011, Holgersson et al., 2022). Pharmacological antipyretics, such as paracetamol and nonsteroidal anti-inflammatory drugs, are commonly administered to reset the hypothalamic set point (Charde et al., 2023, Holgersson et al., 2022, Paul and Walson, 2021). These agents are effective in lowering temperature but may carry risks, including hepatotoxicity, masking of infection, and interference with diagnostic processes (Charde et al., 2023, Franceschi et al., 2023, Paul and Walson, 2021). Non-pharmacological approaches aim to enhance heat loss through conduction, convection, evaporation, or radiation, and include techniques such as tepid sponging, cooling blankets, and fanning (Ariyani et al., 2024, Raak et al., 2022, Sakkat et al., 2021, Widiyanto, 2024). However, these methods may induce shivering, increase metabolic demands, and result in hemodynamic instability, especially in non-sedated or critically ill patients (Raak et al., 2022, Sakkat et al., 2021, Widiyanto, 2024). Despite the risks, many nurses continue to apply these interventions routinely, suggesting a potential disconnect between clinical practice and evidence-based recommendations (Holgersson et al., 2022, Raak et al., 2022).

While previous research has explored nurses’ knowledge and attitudes toward fever, primarily in paediatric contexts(Vicens-Blanes et al., 2021), limited studies have examined how nurses manage fever in adult patients or what underpins their clinical reasoning (Chen et al., 2022, Gouvêa Bogossian et al., 2024), leaving a gap in understanding of how nurses manage fever in adult care and the rationale behind their decisions. Given the critical role of nurses in initiating fever interventions, it is essential to explore the cognitive, institutional, and contextual factors that shape their decision-making.

This study aimed to investigate how nurses manage fever and to examine the rationale underpinning their clinical decisions using a mixed-methods approach. By integrating quantitative and qualitative data, this research seeks to provide actionable insights to inform nursing education, practice guidelines, and evidence-based clinical decision-making. A prior publication (Chen., et al 2022) based on the same dataset primarily reported nurses’ knowledge of fever and their perceptions of fever management. However, it did not explore in depth how this knowledge translated into actual clinical behaviour. The current paper extends beyond knowledge to examine nurses’ decision-making processes and the contextual factors influencing fever management, thereby providing deeper insights and practical implications for nursing education and practice. By so doing, it aims to strengthen the theoretical and practical impact of the findings and contribute to improving patient care in the management of fever.

## 2 Methodology

### 2.1 Study design

This study employed a sequential explanatory mixed-methods design to examine nurses’ approaches to fever management and the rationale underpinning their clinical decisions. A quantitative survey of practice preceded qualitative enquiry to illuminate the mechanisms behind observed patterns. The mixed-methods approach was chosen because practice behaviours (e.g., thresholds for antipyretics, selection of antipyretics methods) are observable at scale, whereas the rationale for these choices is context-dependent and best examined qualitatively (Creswell and Clark, 2011).

To structure integration, we adopted a deductive-to-inductive hybrid analytic stance: quantitative findings and relevant theory informed the qualitative interview guide and initial sensitising concepts (deductive), while thematic development remained open to emergent insights (inductive) (Proudfoot, 2023). Integration occurred at design (sequential), methods (development of the interview guide from survey signals), and interpretation stages (joint displays and meta-inferences). Data on nurses’ knowledge of fever were also collected and have been published separately (Chen et al., 2022), allowing the present study to focus on clinical management of fever and decision-making processes. The design included two phases: a quantitative survey to gather data on clinical practices in fever management, followed by qualitative interviews to explore nurses’ reasoning in greater depth. This mixed-methods approach enabled the integration of descriptive insights with thematic interpretation to provide a comprehensive understanding of clinical decision-making related to fever.

### 2.2 Participants and recruitment

Participants were registered nurses practising in acute and community healthcare settings across Scotland between January 2017 and February 2018. Eligibility required direct involvement in adult patient care and experience managing fever. Recruitment proceeded via institutional mailing lists and professional networks. In total, 177 nurses completed the online questionnaire. Within this survey cohort, 57 respondents provided written reflections to an open-ended question on survey, and 5 volunteers subsequently participated in individual, semi-structured interviews conducted virtually; interviews lasted approximately 30–45 minutes and were audio-recorded and transcribed verbatim.

### 2.3 Data Collection

#### 2.3.1 Quantitative data

The online questionnaire captured demographics (age, gender, education, years in practice, role, clinical specialty/setting), typical patient populations, fever-management strategies (pharmacological and non-pharmacological), and temperature thresholds triggering interventions. We adapted items from previous research (Thompson et al., 2007), originally developed in neuroscience and later reused at scale (Rockett et al., 2015), to fit general adult nursing. Before launching the full survey, we conducted a pilot test to evaluate item clarity, skip-logic, timing, and preliminary psychometrics. A purposive sample of practising registered nurses from acute and community settings (n=30 across specialties) completed the questionnaire and participated in brief cognitive debriefing interviews to identify ambiguous wording and response difficulties. Following that, an expert panel of five nurses (acute/community) assessed content validity and relevance; minor wording changes ensured clarity. The pilot study was conducted again with 30 practising registered nurses in Scotland. This pilot process was to ensure the adapted instrument functioned as intended in routine clinical contexts. Pilot participants were excluded from the main analysis after revisions. Participants were asked to report their preferred strategies for fever management, including both pharmacological approaches, such as the use of paracetamol or nonsteroidal anti-inflammatory drugs, and non-pharmacological methods like tepid sponging or cooling blankets. They were also asked to indicate the body temperature threshold at which they would typically initiate treatment.

To better understand the decision-making process, the questionnaire also explored participants’ primary rationale for initiating antipyretic interventions. Participants were asked to select from predefined options, including adherence to national guidelines, instructions from medical staff, institutional protocols, independent nursing judgement, or other justifications. Using purposive sampling, a total of 177 registered nurses participated in the survey conducted between 2017 and 2018. As no inferential statistical analyses were performed, a priori sample size calculation was not conducted. The sample size was determined based on feasibility and the aim to capture a diverse representation of nursing practices across clinical settings (Polit & Beck, 2021).

#### 2.3.2 Qualitative data

Qualitative data were gathered from two sources. First, open-ended responses provided within the questionnaire offered initial insights into nurses’ thoughts about fever. Second, in-depth data were collected through individual semi-structured interviews with five interviewees who had indicated their willingness to be contacted. The interview guide explored interviewees’ perceptions of fever, their recent clinical experiences managing febrile patients, and the factors that influenced their choices of intervention. All interviews were conducted virtually and lasted approximately 30 to 45 minutes. Recruitment for interviews ceased when data saturation was reached, indicating adequate informational redundancy for the study’s explanatory purpose.

### 2.4 Data analysis

All quantitative analyses were descriptive and were performed in SPSS (Version 25). Participant characteristics and practice patterns are presented as counts and percentages, with the denominator for each item shown in the table where it differs from the total survey sample of 177 due to item non-response. For items that allowed multiple responses (for example, selections across roles, settings, or management strategies), responses were defined as multiple-response sets so that percentages represent the proportion of participants endorsing each option rather than mutually exclusive categories; totals in these rows therefore exceed 100 % by design. Where participants ranked preferred fever-management options, the first, second, and third choices are displayed as separate distributions to convey relative preference order. Categorical fields are shown as frequencies and percentages. Because the study’s quantitative objective was to describe practice patterns rather than to test hypotheses, no inferential statistics are reported in the tables.

For the qualitative data, responses from the open-ended questionnaire item and interview transcripts were analysed using thematic analysis, following the six-phase approach described by Clarke and Braun (2017). To enhance trustworthiness, credibility was supported through independent dual coding of an initial corpus, regular peer-debriefing and consensus meetings to resolve discrepancies, and deliberate attention to disconfirming cases when refining themes (Adler, 2022); dependability was strengthened by maintaining an audit trail of coding decisions, codebook iterations, and analytic memos in NVivo; confirmability was addressed through reflexive memoing, explicit documentation of assumptions, and transparent linkage of exemplar extracts to codes and themes; transferability was facilitated by providing thick description of interviewee roles, settings, and decision contexts (Stahl and King, 2020). Data collection for interviews ceased once no new codes or themes emerged, indicating saturation in relation to the study aim. Member checking with interviewees were not undertaken due to timing constraints, and this limitation is acknowledged in the discussion.

In keeping with the sequential explanatory design, integration occurred at design, methods, and interpretive stages. Quantitative patterns from the survey, such as the frequency of treatment thresholds and the distribution of preferred interventions across settings, were used to shape the interview schedule and sensitising concepts, thereby connecting the strands. During analysis, we constructed joint displays that aligned key proportions with thematically coded explanations from interviews and written reflections, and we used these matrices to develop meta-inferences about why particular strategies were chosen in specific contexts. Where qualitative accounts appeared to diverge from survey patterns, we examined the conditions under which exceptions arose and treated them as explanatory contingencies rather than contradictions, incorporating these clarifications into the final narrative. The integrated interpretation is therefore grounded in observed practice distributions and elaborated by context-rich accounts of reasoning, with the qualitative strand explaining mechanisms underlying the quantitative trends.

### 2.5 Ethical considerations

The study was reviewed and classified as a service evaluation by the Nursing Studies Ethics Panel at the University of Edinburgh, as well as the research governance offices of the participating National Health Service (NHS) boards. As such, full institutional ethical approval was not required. Informed consent was implied through the completion of the online questionnaire, and explicit consent was obtained from all participants involved in follow-up interviews. All responses were anonymised, and data confidentiality was maintained throughout the research process.

## 3 Results

### 3.1 Quantitative findings

A total of 177 registered nurses participated in the quantitative phase of this study. All questionnaires were completed in full, with no missing responses. Additionally, no instances of patterned or non-differentiated responses were identified, suggesting minimal response bias. Participant demographics are presented in Table 1 (Chen et al., 2022). The majority of respondents practised in hospital-based settings (63 %), with diverse roles ranging from staff nurses and charge nurses to nurse practitioners and nurse specialists. Most participants held a bachelor's degree or higher, and their clinical experience spanned a broad range of settings, including medical, surgical, paediatric, and critical care units.

**Table 1:** Demographic data of the participants (*N* = 177) developed from Chen et al., 2022. a. Indicating a multiple choice question. Bachelor of Science (BSc), Bachelor of Nursing (BN), Bachelor of Science (Honours) [BSc (Hons)], Bachelor of Nursing (Honours) [BN (Hons)], Master’s degree (e.g., MSc, MRes, MPhil), and Doctor of Philosophy (PhD).

Description	N( %)
**Setting**	
Hospital Non-hospital	111(62.7) 66(37.3)
**Nursing Role^a^**	
Only with the role of Registered nurse Research related Charge nurse Nurse specialist Nurse practitioner Other Manager	67(37.9) 30(16.9) 24(13.6) 23(13.0) 16(9.0) 12(6.8) 7(3.9)
**Highest educational qualification**	
State Registration Programme Registered General Nurse Diploma Nursing Degree BSc/BN Nursing Degree BSc Hon/BN Hon Post-Graduate Qualification Masters Post-Graduate Qualification PhD	23(13.0) 20(11.3) 50(28.3) 16(9.0) 28(15.8) 1(0.6)
**Experiences in different working unit^a^**	
Acute care Medical Surgical Non-hospital setting Critical care Other Rehabilitation Paediatric Research facility Neuroscience Theatre Psychiatric	100(56.5) 78(44.1) 73(41.2) 71(40.1) 53(29.9) 45(25.4) 29(16.4) 27(15.3) 21(11.9) 20(11.3) 13(7.3) 6(3.4)
**Age (years)**	
21-25 26-30 31-35 36-40 42-45 46-50 51-55 56-60 61-65	5(2.8) 16(9.0) 19(10.7) 26(14.7) 28(15.8) 33(18.6) 29(16.4) 18(10.2) 3(1.7)
**Experience (years)**	
0-5 6-10 11-15 16-20 21-25 26-30 31-35 36-40	24(13.6) 21(11.9) 30(16.9) 23(13.0) 14(7.9) 32(18.1) 22(12.4) 11(6.2)
**Temperature to start fever management (tympanic temperature)**	
37.5°C 37.8°C 38.0°C 39.0°C or above	21(11.9) 12(6.8) 82(46.3) 9(5.1)

About 12 % of participants (*n* = 21) would start to manage fever when the patient’s ear temperature reached 37.5°C, while less than 7 % would start to manage fever (*n* = 12) when the patient’s ear temperature reached 37.8°C. More than 46 % of the participants (*n* = 82) would start to manage fever when the patient’s ear temperature reached 38.0°C, while about 5 % of the participants (*n* = 9) would start to manage fever when the patient’s ear temperature reached 39.0°C or above.

Table 2 presents participants' preferred first-, second-, and third-line choices for fever management, antipyretics were typically prescribed on as-required (pro re nata) basis, with nurses exercising clinical judgement on when to administer within those orders; the preferences reported here therefore reflect nurses’ decisions to administer prescribed on as-required medicine rather than independent prescribing. Pharmacological strategies were most commonly used, with oral or rectal paracetamol selected as the first-line intervention by 45.2 % of participants. Some nurses reported using combined approaches that included environmental cooling (e.g., fans, tepid sponging), although these were less frequently preferred as standalone methods. Ibuprofen (27.1 %) and fan use (14.1 %) were the most frequently cited second-line strategies, while 22 % of participants reported that they would not proceed with further intervention beyond second-line options.

**Table 2:** Preferred choices of fever management interventions.

Type of fever management	N( %)
First choice of fever management	
Paracetamol Oral/Rectal	80(45.2)
Paracetamol Oral/Rectal +Fan in the room	18(10.2)
Fan in the room	7(4)
Paracetamol Oral /Rectal +Tepid sponging	7(4)
Paracetamol Oral /Rectal +Fan in the room+ Tepid sponging	7(4)
Second choice of fever management	
Ibuprofen Oral	48(27.1)
Fan in the room	25(14.1)
Paracetamol intravenous	14(7.9)
Third choice of fever management	
None	39(22)
Fan in the room	27(15.3)
Tepid sponging	26(14.7)
Primary rationale for initiating fever management	
Independent professional judgement	87(49.2)
National guideline	28(15.8)
Medical direction	27(15.3)
Unit protocol	21(11.9)
Other	14(7.9)

When asked about the rationale for initiating fever management, nearly half of participants (49.2 %) cited independent nursing judgement. Other reasons included adherence to national guidelines (15.8 %), following medical direction (15.3 %), unit protocols (11.9 %), and unspecified “other” reasons (7.9 %).

### 3.2 Qualitative findings

Five nurses participated (four women, one man), ranging in age from 26 to 65 years. Roles included staff/registered nurse (n=2), research nurse (n=2), and nurse specialist (n=1). Years of nursing experience were 0–5 years (n=3), 16–20 years (n=1), and 26–30 years (n=1). Current practice settings comprised acute care (n=1), a medical unit (n=1), a psychiatric unit (n=1), and non-hospital settings (n=2). Several interviewees reported prior experience spanning critical care, acute care, surgical, and psychiatric units, which informed their approaches to fever management.

Thematic analysis of the qualitative data generated from open-ended questionnaire responses and semi-structured interviews revealed five major themes with associated subthemes: (1) Clinical Routine and Intuition; (2) Sepsis Framing, Fever Phobia, and Risk Perceptions; (3) Comfort and Patient-Centred Drivers; (4) Knowledge Gaps and Uncertainty; and (5) Limited Critical Reflection. These themes are elaborated in the following sections.

### 3.3 Theme 1: clinical routine and intuition

Interviewees frequently described their fever management strategies as routine and largely driven by clinical habit or ward culture. Interventions such as administering paracetamol or removing excess clothing were performed as default actions, often without reconsideration of their appropriateness or alignment with evidence-based recommendations. Many nurses expressed that the immediate response to fever was to apply a fan, remove blankets, or initiate pharmacological antipyretics, despite their awareness that some of these approaches, such as tepid sponging, are no longer supported in current guidelines. In addition, several nurses described adapting or simplifying their fever management when faced with competing clinical tasks, such as managing multiple acutely unwell patients or time-critical procedures. In these situations, they reported prioritising rapid, familiar interventions (for example, administering paracetamol alone) and postponing or omitting additional non-pharmacological measures.

*"That is my way; it’s really just the tepid sponging and making sure that they are cooled down by removing clothes and things. As well as that, there’s the paracetamol and ibuprofen—really that’s it." (Interviewee A)*

These routine practices were often intuitive rather than deliberative. As one interviewee put it:

*"You do it in a very systematic way. You do it without thinking." (Interviewee G)*

This automation may contribute to the continuation of outdated practices, such as physical cooling methods that may induce shivering and increase metabolic demand. Physical antipyretics such as fans and damp cloths were often applied for comfort purposes rather than for clinically reducing temperature. One participant shared:

*"Whilst tepid sponging is not recommended, I would advise using a cool, damp cloth to wipe away sweat from face for comfort reasons occasionally." (Participant 39, questionnaire)*

### 3.4 Theme 2: sepsis framing, fever phobia, and risk perceptions

Nurses consistently linked fever to infection and, more critically, to sepsis—a framing that prompted immediate and often aggressive management strategies. Most interviewees described a clinical environment where fever was seen not merely as a symptom but as a signal of potential systemic collapse. This framing reflected a shift from considering fever as a self-limiting physiological response to viewing it as a diagnostic trigger for sepsis intervention. Consequently, fever management was frequently bundled with the initiation of the Sepsis Six protocol, a United Kingdom bedside care bundle (Sepsis Trust) comprising timely oxygen administration, blood cultures before antibiotics, prompt intravenous antibiotics, measurement of serum lactate, intravenous fluid resuscitation, and close monitoring of urine output, thereby embedding antipyretic decisions within a broader early-sepsis response. As shown in Table 2, 15.8 % of participants reported using national guidelines to inform decisions about fever management. Among these respondents, more than 70 % specifically cited the Sepsis Six protocol.

"Within an hour you should be giving paracetamol, you should be giving IV fluids. You should be giving IV antibiotics. You should be taking blood cultures..." (Interviewee M)

*"Fever is managed once patient has been assessed and antipyretics are usually accompanied by IV antibiotics as per policy." (Participant 40, questionnaire)*

This interpretation of fever led nurses to rely heavily on antibiotics and fluids, sometimes even in the absence of a confirmed infection. Several nurses acknowledged that such measures were standard within institutional protocols. However, they also recognised the risk of assuming all fevers were infectious in origin.

*"Which I normally treat with antibiotics … paracetamol and ibuprofen. There used to be a lot more antibiotics given out for fevers and things like that." (Interviewee A)*

This strong infection-focused response, although clinically relevant in cases of confirmed sepsis, often overshadowed other differential diagnoses and may have contributed to overtreatment. Accompanying this infection framing was a deep-seated fear of the potential consequences of fever, reinforcing a culture of rapid intervention. Interviewees expressed concerns that uncontrolled fever could lead to febrile seizures, organ failure, or even death.

*"It can cause kidney damage and things like that, so we’re trying to avoid major organ shutdowns." (Interviewee A)*

*"Fever is an indication that you’ve something way more serious going on here... people can die in delay from sepsis." (Interviewee G)*

These exaggerated perceptions of risk, commonly referred to in the literature as “fever phobia,” (Crocetti et al., 2001, Merlo et al., 2023, Purssell and Collin, 2016) were evident throughout the interviews and open-text survey responses. Nurses frequently overestimated the risks of untreated fever and underestimated its physiological benefits, despite some expressing awareness of more nuanced views. Notably, this fear was reinforced through the structure of clinical care, especially by the widespread implementation of the Sepsis Six protocol. While the protocol appropriately prioritises rapid treatment in cases of suspected sepsis, its strict algorithmic nature appeared to reinforce an automatic response to fever, treating it as an urgent precursor to deterioration. As a result, nurses often viewed fever not as a symptom to monitor but as an alarm bell necessitating full sepsis protocol activation. This mechanistic framing left little room for nuanced clinical judgement, with several participants expressing uncertainty about when the Sepsis Six protocol should or should not be applied. The convergence of institutional expectations, protocol pressure, and underlying clinical fear may thus contribute to persistent overtreatment of fever and resistance to adopting more conservative, evidence-based approaches.

*"The use of antipyretics will not prevent febrile convulsions; a large majority of nurses in my experience are very fever phobic." (Participant 31, questionnaire)*

Interestingly, only a minority of participants acknowledged the immunological benefits of fever. Some recognised that the body’s febrile response could assist in combating infection by promoting leukocyte activity, but this knowledge rarely influenced their clinical choices.

*"I know that there is literature around the fact that when patients have a temperature, that’s actually quite a good thing because it’s dealing with the infection..." (Interviewee M)*

### 3.5 Theme 3: comfort and patient-centred drivers

While national guidelines recommend treating fever primarily to relieve discomfort, the qualitative data suggested that the extent to which nurses assessed patient comfort before initiating fever management varied. Although many participants cited comfort as a reason for intervention, the decision to act was often influenced more by the presence of fever itself than a clear indication of patient distress. Interventions such as administering paracetamol or removing layers of clothing were frequently applied pre-emptively, sometimes without explicit confirmation of discomfort.

There were also indications that comfort-based interventions were guided by anticipated rather than reported symptoms. In some instances, patients requested antipyretics, which nurses interpreted as a cue for action. Yet, the decision-making process appeared influenced as much by routine and perceived expectations as by patient-reported need.

*"And you find a lot of the time our patients will come and ask for it… ‘Oh I have a cold’, ‘I have flu’, ‘I’m not feeling great’, ‘I think I need some paracetamol’." (Interviewee G)*

*"While not wanting to suppress antibody production, analgesics such as paracetamol may be given to promote comfort." (Participant 38, questionnaire)*

Non-pharmacological measures, such as encouraging oral fluids or using fans, were often used alongside medications. Interestingly, comfort was often cited as a justification even when clinical markers did not necessarily support intervention. For example, some participants administered paracetamol or initiated cooling measures without revisiting the patient’s comfort level post-intervention. This routine was particularly evident in the early stages of applying sepsis protocols. While comfort appeared to be a central motivation, there was limited discussion among participants of evaluating the patient’s comfort level after intervention. In practice, comfort-driven decision-making seemed to operate within broader institutional habits and expectations. As such, it may have served both as a sincere concern and as a post hoc rationalisation for initiating familiar treatments in response to fever.

### 3.6 Theme 4: knowledge gaps and uncertainty

Participants acknowledged inconsistencies in their knowledge about fever physiology and management. Many admitted to being unsure of the rationale behind their actions and expressed confusion or concern regarding best practices.

*"I feel uncomfortable with the lack of clarity surrounding this issue." (Participant 12, questionnaire)*

*"I wasn’t 100 % sure that my care was the same as everybody else’s." (Interviewee A)*

Although some participants had a strong awareness of fever’s benefits, many lacked confidence in their knowledge and relied on clinical routines rather than evidence-based guidance.

In addition to expressing uncertainty, several nurses described consciously slowing down their decision-making when managing febrile patients. Rather than acting automatically, they reported pausing to review trends in vital signs, reconsider whether an elevated temperature might be adaptive, and, at times, delaying antipyretic administration to avoid obscuring important diagnostic information. These accounts illustrate instances of more reflective, analytic reasoning about fever management, beyond routine practice.

### 3.7 Theme 5: limited critical reflection

While some interviewees demonstrated awareness of the knowledge-practice gap in fever management, few described engaging in meaningful critical reflection that translated into changes in behaviour. Many appeared to rely on habitual practices or guidance from colleagues without questioning whether these approaches were consistent with the latest evidence. Even when gaps in knowledge were recognised, these were not always perceived as sufficient reason to modify clinical routines.

*"I haven’t felt the need to go and look it up… never even questioned it." (Interviewee C)*

Some interviewees described their clinical decisions as intuitive or based on what was commonly done in the ward rather than on critical appraisal of the evidence. This reliance on routine may have limited opportunities to evaluate the rationale behind specific interventions or to assess whether fever required treatment in the first place.

*"It’s almost like a processing line; people get packaged up and everything that needs to be done for them gets done. People are very practiced at what happens." (Interviewee M)*

The comfort of following familiar practices, paired with a lack of formal prompts for reflection, may have contributed to a reduced sense of urgency around updating one’s knowledge or challenging embedded norms. In some cases, this hesitancy was linked to limited confidence in clinical reasoning, particularly among less experienced staff.

*"I guess you also doubt yourself as to whether you have enough experience or knowledge?" (Interviewee R)*

Together, these findings suggest that while awareness of best practices may exist, the ability to question, reflect, and adapt is constrained by institutional culture, confidence levels, and a lack of time or space for critical discussion. This highlights the need for supportive structures that enable reflective practice and promote confidence in evidence-based decision-making.

**Fig. 1:**
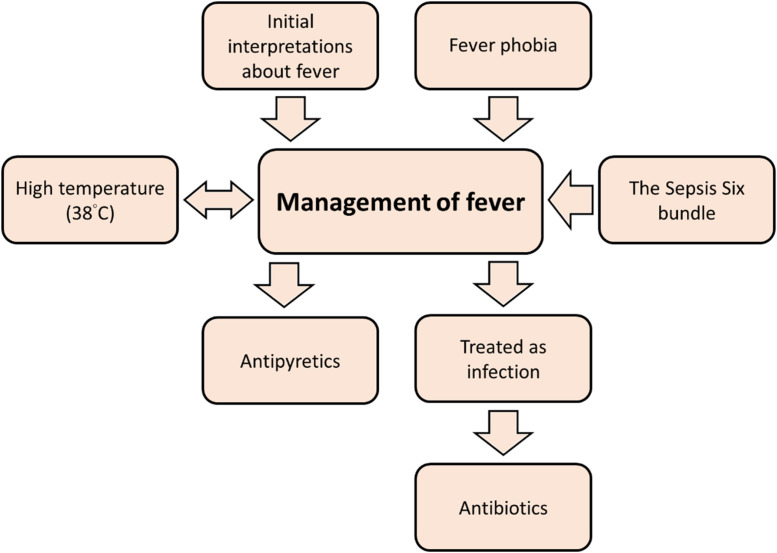
Factors relating to fever management. The direction of the arrows indicates the direction of influence. An arrow that points in both directions indicates that the two factors can influence each other.

To frame the behavioural mechanisms underpinning our findings, Fig. 1 maps the principal factors that shape fever management and the directionality of their influence. Fig. 1 depicts a network in which institutional protocols, sepsis framing, perceived risk, clinical habit, and assessments of patient comfort interact to shape nurses’ decisions about fever management. Bidirectional arrows indicate reciprocal reinforcement, for example between protocol pressure and risk-averse practice, while unidirectional arrows denote dominant causal flow, such as from routine ward culture to default antipyretic use.

## 4 Discussion

This mixed-methods study advances understanding of nurses’ fever management by bringing the survey distributions and the qualitative accounts into a single, integrated interpretation. The quantitative strand established the contour of practice, for example, the predominance of pharmacological strategies and the ranking of paracetamol as a first-line choice, while the qualitative strand explained the rationale of decision-making that made those patterns likely. Nurses’ narratives situated prescribed on as-required antipyretic administration within authorised orders and described how bedside assessment, perceptions of patient risk, and workload shaped the decision to administer or defer, thereby clarifying that the “choice” captured in the survey represents nursing judgement within prescriber-authorised parameters rather than independent prescribing. The lower frequency of non-pharmacological strategies observed in the survey was likewise explained qualitatively: interviewees framed these measures as adjuncts that were selectively used when patients were uncomfortable or when antipyretics were contraindicated, but often deprioritised when early-sepsis workups were underway.

Integration was particularly evident in the alignment between the survey’s temperature-threshold patterns and interview accounts linking fever to sepsis risk. Quantitative findings showed a concentration of treatment thresholds in a narrow band, and the qualitative data illuminated why: many interviewees described fever as an early warning for systemic deterioration and reported embedding antipyretic decisions within the Sepsis Six response, which prioritises antibiotics, fluids, haemodynamic assessment, and cultures. Where strands diverged, the qualitative material provided conditional explanations. For example, respondents who reported not proceeding beyond a second-line option often did so, by their account, because clinical reassessment suggested that comfort had improved or that the clinical trajectory mandated escalation to sepsis pathways rather than additional antipyresis. These contingencies indicate that apparent inconsistencies across strands reflect context-sensitive decision rules rather than random variation.

Taken together, the strands support three meta-inferences. First, nurses’ fever management is best understood as situated reasoning enacted within as required authorisations and institutional protocols, rather than as a simple preference for one intervention over another. Second, sepsis-oriented framing exerts a strong organising influence on practice, narrowing thresholds and coupling antipyretic with early-sepsis bundles. This helps explain the prominence of pharmacological first-line treatment in the survey data. Third, variability in second- and third-line actions is not merely idiosyncratic but appears responsive to patient condition, perceived trajectory, and competing clinical tasks. Quantitatively, nurses’ second- and third-line choices did not converge on a single “standard” response but were distributed across pharmacological and non-pharmacological options (for example, ibuprofen 27.1 %, fan in the room 14.1 %, and intravenous paracetamol 7.9 % as second-line choices; no further action 22.0 %, fan in the room 15.3 %, and tepid sponging 14.7 % as third-line choices), suggesting that nurses adapt their actions to contextual demands rather than following a fixed sequence. Qualitative accounts in Theme 1 further indicated that nurses adjusted or curtailed interventions in response to the patient’s overall condition and to competing clinical priorities, particularly when workload was high or when other patients required urgent attention. These integrated interpretations have practical implications for education and guideline implementation: teaching should emphasise how to calibrate prescribed on as-required administration within sepsis pathways, when non-pharmacological measures add value, and how to document the clinical reasoning that links assessment to action. Finally, the integration acknowledges the descriptive scope of the quantitative sample and uses the qualitative strand to avoid over-interpretation of subgroup differences, thereby strengthening the credibility and usefulness of the conclusions.

This study offers a nuanced examination of how nurses approach fever management, shedding light on the interplay between individual judgement, institutional protocols, and tacit clinical norms. Rather than merely cataloguing practices, the findings reveal a more fundamental challenge: the habitual, often unexamined, nature of clinical decisions concerning fever. These insights carry implications not only for nursing practice but also for how clinical reasoning is cultivated and supported in everyday care.

### 4.1 Revisiting decision-making through the dual process lens

**Fig. 2:**
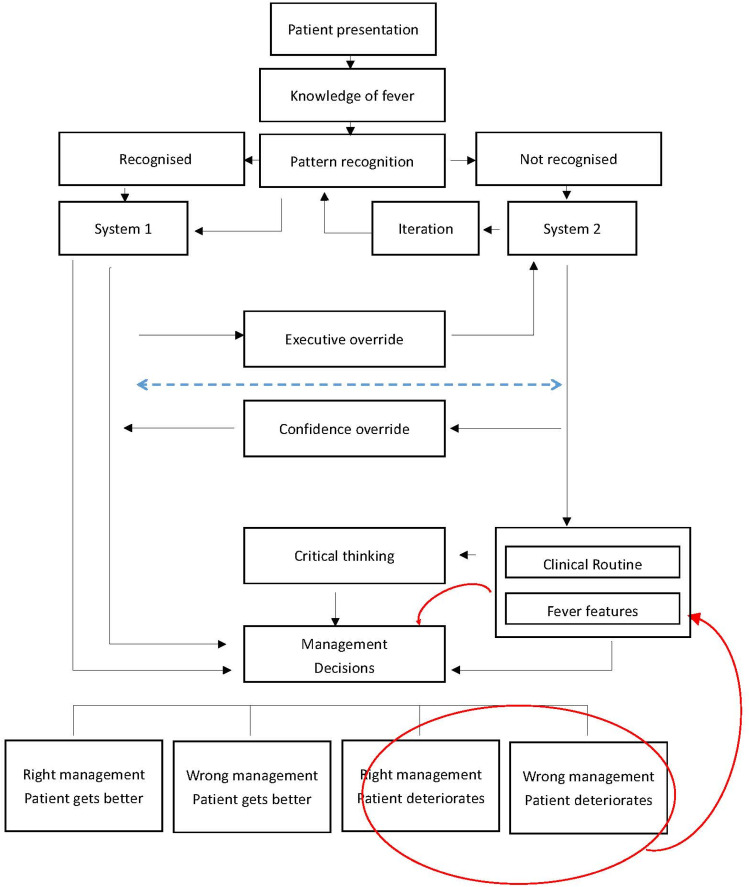
The dual process theory of decision making in the context of fever adapted from Croskerry (2005) (Croskerry & Nimmo, 2011). System 1 reflects fast, automatic, pattern-based judgments that are often necessary and effective. System 2 reflects slower, analytical reasoning that supports critical appraisal and guideline-aligned decisions. The dashed lines indicate little to no engagement of System 2 along those pathways. In our study, when patients appeared to deteriorate (red circle), nurses often intuitively attributed the change to fever (red upward arrow) and intensified antipyretic treatment (red downward arrow), reinforcing the belief that fever requires active management.

Dual process theory proposes two modes of reasoning in clinical practice. System 1 refers to fast, automatic, and pattern-based judgement that is efficient but vulnerable to bias. System 2 refers to slower, analytical, and deliberative reasoning that supports critical appraisal and guideline concordance. In this study, fever management was largely governed by System 1 processes. Themes 2, 4, and 5 indicate that nurses frequently acted intuitively, often coupling fever with presumed infection and early sepsis responses, despite uncertainty and limited critical reflection (as presented in Fig. 2)(Croskerry and Nimmo, 2011). However, some nurses also described moments of deliberate “stopping to think” as discussed in Theme 4 (Knowledge Gaps and Uncertainty), for example, pausing to review trends in vital signs or reconsidering whether an elevated temperature might be adaptive. In contrast, contexts characterised by high workload, strong protocol pressures, or entrenched ward culture are shown in the Fig. with dashed lines, indicating that System 2 engagement was minimal or absent along those pathways. In our study, when patients appeared to deteriorate (red circle), nurses often intuitively attributed the change to fever (red upward arrow) and reinforced antipyretic management (red downward arrow), further strengthening the perception that fever should be actively treated. While intuitive (System 1) reasoning has often been criticised for contributing to clinical error, our findings suggest that these automatic responses are not formed in isolation. Instead, intuition in fever management appears to be shaped by repeated exposure to standardised responses, such as the routine application of antipyretics or default activation of the Sepsis Six. Over time, these actions become embedded as automatic responses, making them appear instinctive even when they originate from formal protocols.

This raises important questions about the distinction between clinical intuition and institutionalised habit. If what appears to be professional judgement is, in fact, the residue of protocol adherence, then efforts to promote critical reasoning must look beyond individual cognition and address the systems that shape practice. As there is not yet a dedicated fever management protocol, our data suggest that fever often functions as a trigger for sepsis pathways, reflecting a heuristic that equates “fever = infection,” even though only about half of fevers were infection-related in our sample (Fig. 1). Accordingly, system-level adjustments are warranted: (i) refine sepsis-pathway entry criteria so that fever alone is not a sufficient trigger; (ii) embed a brief diagnostic “pause point” to prompt review of vital-sign trajectories, differential diagnoses (including non-infectious causes), and red flags; (iii) introduce a concise fever assessment aid for clinically stable patients that prioritises comfort-focused measures and observation with clearly signposted escalation criteria; and (iv) implement audit-and-feedback on sepsis bundle activations (e.g., proportion initiated for fever alone, subsequent diagnoses, and outcomes) to curb over-activation. These organisational interventions align decision pathways with evidence and reduce reliance on the “fever to sepsis” shortcut.

### 4.2 The hidden risks of ‘safer’ decisions

Another insight concerns the implicit risk calculus that appears to guide nursing behaviour. Despite knowledge of the physiological role of fever and the guidance from the National Institute for Health and Care Excellence to treat only when discomfort is present (NICE, 2019), nurses in this study often inclined towards intervention. This tendency may stem from what could be termed “defensive care”, where action is favoured over inaction due to the fear of missing deterioration, even when evidence does not support intervention (Lorenc et al., 2024, Raposo, 2019). Such practice patterns reflect a culture in which overtreatment is seen as the safer or more professional choice.

This instinct to “do something” in response to fever may be reinforced by the invisibility of harm. The adverse consequences of overtreatment, such as diagnostic delay or antimicrobial resistance, are diffuse and delayed, making them harder to perceive compared to the immediate risks of non-intervention (Aronoff and Neilson, 2001, Ludwig and McWhinnie, 2019, Zimmermann and Curtis, 2017). As such, the decision to initiate treatment may be as much about mitigating perceived liability or discomfort with clinical ambiguity as it is about alleviating patient symptoms.

### 4.3 Knowledge alone is not enough

While participants generally held positive beliefs about controlling fever (Chen., et al 2022), these beliefs were not predictive of their actual decision-making. This disconnect suggests that educational interventions focusing solely on knowledge enhancement may be insufficient (Clarke et al., 2021, Pursio et al., 2021, Thompson et al., 2004). As long as institutional norms and clinical cultures tacitly endorse certain practices, even well-informed clinicians may struggle to act differently (Barrott et al., 2023, Clarke et al., 2021, Pursio et al., 2021). What is needed, therefore, are educational strategies that address not only knowledge deficits but also the contextual and emotional drivers of care, such as fear, uncertainty, and normative expectations (Alharbi et al., 2024, Graham et al., 2018, Pfeffer and Sutton, 1999, Ward et al., 2009).

Encouragingly, some participants demonstrated a willingness to question routine care, especially when prompted by external feedback or structured reflection. This points to a promising role for facilitated discussion, interprofessional debriefs, and the use of case-based learning to promote reflective practice and challenge embedded habits.

### 4.4 Clinical implications

To promote more evidence-informed and person-centred approaches to fever management, this study underscores the need for multifaceted interventions that go beyond knowledge enhancement. This conclusion is supported by three findings. First, Themes 2, 4, and 5 show a predominance of intuitive responses in which fever was routinely linked to infection and potential sepsis, with rapid initiation of antipyretics or sepsis pathways and limited explicit appraisal of alternatives. Second, only 15.8 % of participants reported using national guidelines to inform decisions, and most of these respondents referred to the Sepsis Six protocol, indicating protocol activation rather than critical interpretation of guideline intent (Table 2). Third, Fig. 1 maps how protocol pressure, ward routines, perceived risk, and clinical habit channel decisions toward System 1 and constrain deliberative System 2 reasoning. Taken together, these findings indicate that effective change will require both cognitive and organisational supports: below, we outline complementary strands targeting clinicians’ reasoning and the systems that shape practice.

It is essential to enhance nurses’ contextual awareness by encouraging them to reflect not only on clinical guidelines but also on the situational and cultural factors that influence their decision-making (Pursio et al., 2021, Thompson et al., 2004). Training should make explicit how habitual practices and institutional expectations shape responses to fever, and prompt consideration of alignment with patient-centred goals. Fostering reflective capacity within teams is likewise crucial. Structured opportunities, such as post-shift debriefings, case-based discussions, or interprofessional dialogues, can help clinicians appraise practice, question ingrained routines, and weigh alternatives (Razieh et al., 2018, Truglio-Londrigan and Slyer, 2018). Educational content should also reframe risk: alongside the harms of under-treatment, the subtler risks of over-treatment (e.g., antimicrobial resistance, diagnostic masking) should be salient to decision-makers (Razieh et al., 2018). Finally, while intuitive (System 1) responses are intrinsic to practice (Croskerry and Nimmo, 2011), their accuracy depends on regular recalibration through critical reflection, updated evidence, and team dialogue (Nibbelink and Brewer, 2018, Razieh et al., 2018, Truglio-Londrigan and Slyer, 2018). By addressing both the cognitive and contextual dimensions of decision-making, these strategies can help to narrow the gap between evidence and everyday nursing practice (Johansen and O'Brien, 2016, Ward et al., 2009).

### 4.5 Limitation of the study

A principal limitation is temporal. Data were collected in Scotland between January 2017 and February 2018, that is, after publication of the National Institute for Health and Care Excellence guideline on suspected sepsis (2013), but before the publication and wider dissemination of the updated National Institute for Health and Care Excellence’s guideline on fever in children under 5 years (November 2019, which replaced the 2013 version). Although our study concerns adult nursing practice, the paediatric fever guideline is often cited in institutional education and may shape general perceptions of fever; consequently, our dataset reflects early implementation of the National Institute for Health and Care Excellence’s 2013 guideline in routine services, with inevitable variation in local dissemination and uptake across organisations. The study also precedes the COVID-19 pandemic, when the salience of fever as a potential marker of transmissible viral illness, and its linkage to isolation, testing, and infection-prevention protocols, intensified. As such, participants’ perceptions, clinical priorities, and decision-making processes may have reflected a pre-pandemic understanding of fever that differs markedly from current practice. In the post-COVID era, fever has gained heightened clinical and public significance, not only as a symptom warranting isolation or testing, but also as a trigger for infection control measures and public health interventions. This evolving landscape raises important questions about how nurses’ responses to fever may have shifted in response to pandemic-related policies, heightened vigilance, and broader societal awareness. Future research is needed to explore how the meaning and management of fever have changed in light of COVID-19 and whether such shifts have led to more reflective or, conversely, more defensive clinical practices.

## 5 Conclusion

This study highlights how nurses’ fever management practices are shaped by deeply embedded routines and institutional frameworks, particularly the influence of the Sepsis Six protocol and widespread fever phobia. While nurses often acted on perceived clinical urgency, such responses were not always grounded in patient discomfort or evidence-based rationale. These findings underscore the need for greater emphasis on reflective practice, critical appraisal of protocols, and education that addresses both clinical knowledge and the emotional drivers of overtreatment.

Data Statement

Due to the sensitive nature of the questions asked in this study, survey respondents were assured that raw data would remain confidential and would not be shared.

## Funding

This study did not receive any funding

## CRediT authorship contribution statement

**Lu-Yen A Chen:** Writing – review & editing, Writing – original draft, Validation, Project administration, Methodology, Investigation, Formal analysis, Conceptualization. **Tonks N Fawcett:** Writing – review & editing, Writing – original draft, Validation, Supervision, Methodology, Formal analysis, Conceptualization. **Colin Chandler:** Methodology, Formal analysis. **Tzu-Wen Weng:** Writing – review & editing.

## Declaration of competing interest

The authors declare that they have no known competing financial interests or personal relationships that could have appeared to influence the work reported in this paper.
